# Recent Discovery of Diverse Prophages Located in Genomes of *Vibrio* spp. and Their Implications for Bacterial Pathogenicity, Environmental Fitness, Genome Evolution, Food Safety, and Public Health

**DOI:** 10.3390/foods14030403

**Published:** 2025-01-26

**Authors:** Yafei Ou, Jun Yan, Yongjie Wang, Lanming Chen

**Affiliations:** 1 Key Laboratory of Quality and Safety Risk Assessment for Aquatic Products on Storage and Preservation (Shanghai), Ministry of Agriculture and Rural Affairs of China, Shanghai 201306, China; 2 College of Food Science and Technology, Shanghai Ocean University, Shanghai 201306, China

**Keywords:** *Vibrio*, prophage, virulence, environmental fitness, genome evolution, food safety

## Abstract

Bacteria in the genus *Vibrio*, including at least 152 species, thrive in marine and estuarine environments and are frequently detected in aquatic products worldwide. Of these, 12 species have been implicated in human infectious diseases, such as the life-threatening pandemic cholera, acute gastroenteritis, and severe sepsis. Nevertheless, molecular mechanisms of their pathogenesis are not fully uncovered yet. Prophages are found prevalent in *Vibrio* spp. genomes, carrying a number of genes with various functions. In this review, we deciphered the evolutionary relationship between prophages and *Vibrio* species and highlighted the impact of prophages on the bacterial pathogenicity, environmental fitness, and genome evolution, based on 149 newly discovered intact prophages located in the genomes of 82 *Vibrio* spp., which we searched and collected from Web of Science Core Collection in the most recent 5 years. The effects of prophages on resistance to superinfection, strain competition, and their regulation were also discussed. This review underscored crucial roles of prophages in shaping *Vibrio* spp. genomes and their implications for food safety and public health.

## 1. Introduction

The genus *Vibrio* contains a large subgroup of bacteria in the family *Vibrionaceae*, the order *Vibrionales*, and the class *Gammaproteobacteria*. They thrive in marine and estuarine environments and are found in fish, corals, shrimps, plankton, and mammals [1,2]. For instance, most recently, Zeidler et al. [3] surveyed the *Vibrio* spp. prevalence in seafood samples (*n* = 306) including shrimp and mussel collected in Berlin, Germany from March 2023 to January 2024. Their results showed that *Vibrio* spp. were detected in 56% of the samples, among which *Vibrio parahaemolyticus* (58%) was the most prevalent species, followed by *Vibrio alginolyticus* (42%), *Vibrio cholerae* non-O1/non-O139 (25%), and *Vibrio vulnificus* (4%) [3]. These bacteria are generally able to grow on marine agar and selective thiosulfate-citrate-bile salt-sucrose (TCBS) agar [4]. The genus *Vibrio* consists of at least 152 species (https://lpsn.dsmz.de/genus/vibrio, accessed on 10 August 2024), of which 12 species have been linked to human infectious diseases, including *V. alginolyticus*, *Vibrio cincinnatiensis*, *V. cholerae*, *Vibrio damsela*, *Vibrio fluvialis*, *Vibrio furnissii*, *Vibrio harveyi*, *Vibrio hollisae*, *Vibrio metschnikovii*, *Vibrio mimicus*, *V. parahaemolyticus*, and *V. vulnificus* [5].

*V*. *cholerae*, *V*. *parahaemolyticus*, and *V*. *vulnificus* are the most prominent species causing infectious diseases in humans, followed by *V*. *alginolyticus* [6]. Of these, *V. cholerae* can cause cholera, being the most terrifying pandemic diarrhea disease worldwide, usually transmitted via contaminated food or water [7]. Since 1817, seven cholera pandemics have been recorded, among which the first six were caused by *V. cholerae* of the classical biotype, and the seventh, caused by the El Tor biotype, has been ongoing since 1961 [8]. Approximately 1.3 to 1.4 million people in the world are infected with cholera each year, causing 21,000 to 143,000 people to die (www.cdc.gov/cholera/index.html, accessed on 21 September 2024). Most recently, the 2022–2023 cholera outbreaks led to 59,156 cases and 1771 deaths in Malawi [9]. Recently, Chen et al. [10] were the first to report experimental evidence for potentially toxic *V*. *cholerae* in snails. They isolated *V. cholerae* strains (*n* = 203) from 36 species of aquatic food animals, nearly two-thirds of which had not been tested before. Although no isolate was found to carry cholera toxin genes *ctxAB* (0.0%), the isolates were found to be positive for virulence genes *tcpA* (0.98%), *ace* (0.5%), and *zot* (0.5%), which derived from *Cipangopaludina chinensis*. High percentages of virulence-associated genes were also noted, including *hapA* (73.4%), *rtxCABD* (68.0–41.9%), *tlh* (54.2%), and *hlyA* (37.9%) [10]. Most recently, Yan et al. [11] demonstrated that different secretomes and proteomes of *V. cholerae* isolates were triggered by the matrices of eight species of edible aquatic animals, which facilitated the bacterial resistance in the aquatic animals and increased its pathogenicity to the host [11].

Vibriosis is a group of clinical syndromes associated with non-cholera *Vibrio* spp., such as gastroenteritis, septicemia, and invasive skin and soft tissue infections [5]. For instance, *V. parahaemolyticus* is a top seafood-borne pathogen in the world and can cause acute gastroenteritis, septicemia, and even death [12]. Increased *V. parahaemolyticus* contamination has been reported in processed, ready-to-eat foods, implying that this bacterium can survive in the harsh circumstance of food processing [13]. For example, most recently, Wu et al. [14] collected 154 crayfish (*Procambarus clarkii*) and environmental samples from crayfish farms and wholesale and retail markets from May to September 2024 in Jiangxi Province, China. They found that *V. parahaemolyticus* was detected in 66% of the crayfish production–sale chain, and in 92% of the market samples. Crayfish tanks contributed to the highest contamination percentage. The crayfish surface, which could be adsorbed by 90% of the *V. parahaemolyticus* cells in 6 h, was more susceptible to bacterial contamination than the crayfish intestine [14]. *V. vulnificus* can infect open wounds through contact with saltwater, brackish water, or raw seafood, causing sepsis and cellulitis [15,16]. It occurs in high numbers in seafood such as marine fish, shrimps, oysters, and crabs around the world, particularly in warmer months [17]. *V. alginolyticus* is the etiologic agent of severe soft tissue infections, sepsis, and other extraintestinal infections [18]. For instance, Abdelsalam et al. [19] reported a disease outbreak featured by caligid copepod infestations and subsequent *V*. *alginolyticus* infections in European seabass (*Dicentrarchus labrax*) and flathead grey mullet (*Mugil cephalus*) grown at a private facility in Deeba Triangle, Egypt. Moreover, the *V. alginolyticus* isolates showed resistance to β-lactams, aminoglycosides, and trimethoprim-sulfamethoxazole [19]. Recently, Sun et al. [20] collected 128 *V*. *alginolyticus* isolates across China in 2020, which were isolated from seafood (*n*  =  75), freshwater products (*n*  =  51), and other samples (*n*  =  2). They found that 95.31% of the *V. alginolyticus* isolates were resistant to at least one antibiotic category. Fifteen virulence genes were present in *V. alginolyticus* isolates (*n* = 57), such as the type III secretion system (T3SS) translocated factors-related genes *vopD*, *vopB*, and *vcrH* (54.4%, 31/57), T3SS-regulated gene *tyeA* (54.39%), and T3SS-inner rod protein and needle protein genes *vscI* and *vscF* (50.88%) [20]. The results highlighted the prevalence of *V. alginolyticus* in both freshwater and seafood products. It is estimated that 80,000 cases of human vibriosis occur in the United States each year (www.cdc.gov/vibrio/index.html, accessed on 21 September 2024). The rising incidence of vibriosis is being reported globally. Most recently, Morgado et al. [21] analyzed vibriosis case data for Maryland (2006–2019; *n* = 611), USA, obtained from the Cholera and Other *Vibrio* Illness Surveillance (COVIS) system managed by the Centers for Disease Control and Prevention (CDC). They found a 39% (*p* = 0.01) increase in the average annual incidence rate (per 100,000 population) of vibriosis, comparing the 2006–2012 and 2013–2019 periods. *V. vulnificus* infections showed the highest percentage increase (53%, *p* = 0.01), followed by *V. parahaemolyticus* (47%, *p* = 0.05). The long-term increases in *Vibrio* infections are happening in Maryland, together with enhanced rates of hospitalization and average hospital durations [21]. Thus, a better understanding of the pathogenetic underpinnings of these *Vibrio* spp. should have profound implications for the effective control and prevention of cholera and vibriosis.

Phages, being the most abundant biological entities in the biosphere, are viruses that infect bacteria [22]. After infecting bacterial hosts, virulent phages proliferate in large numbers, lyse host cells, and release progeny phage particles. The life cycle of temperate phages includes a lysogenic conversion in which phages insert into the host chromosomes and become prophages [23]. Studies have indicated that prophages vary in bacterial genomes and can endow their hosts with new biological functions through their carried genes [24,25,26]. Most recently, Steensen et al. [27] reported that tailless and filamentous prophages are very prevalent in marine *Vibrio*, and proposed that these prophages are active and highly rich drivers of host ecology and evolution, like the well-known tailed ones [27]. Virulence genes in prophages can be transmitted at high frequencies by horizontal gene transfer (HGT), resulting in the emergence of pandemic, epidemic, or pathogenic clones of *Vibrio* spp., thus posing a high risk to food safety and public health [28,29,30].

To the best of our knowledge, complete genome sequences of 557 phages hosted by *Vibrio* spp. have been deposited in the Viral Genome Resource at National Center for Biotechnology Information (NCBI) (https://www.ncbi.nlm.nih.gov/labs/virus/vssi, accessed on 27 August 2024). By searching the Web of Science Core Collection database, we collected 149 intact prophage gene clusters identified in the genomes of 82 *Vibrio* isolates in the most recent 5 years (Table S1). In this review, we focused on these newly discovered prophages in *Vibrio* spp., specifically, their impact on the pathogenicity, environmental fitness, and genome evolution of *Vibrio* spp.

## 2. Materials and Methods

### 2.1. Data Source and Searches

In this review, the Web of Science Core Collection database was used to collect the literature. The search terms used were as follows: “Vibrio” and “prophage”. The publications were retrieved from 1 January 2020 to 19 September 2024. The inclusion criteria of the review were met if articles addressed the *Vibrio* spp., prophages, genome features, resistance, virulence, transmission, evolution, or regulation. Moreover, the studies’ origin, sample size, strain origin, strain identification, sequencing technique, and number of prophages were also assessed. Furthermore, the information in the titles and abstracts was assessed, and the full texts were examined. The exclusion criteria were as follows: studies conducted on non-named strains; research objectives that focused on *Vibrio* genomes without prophages; studies aimed at the application of phages but that did not have any of the above results; and publications that were not in English. The searches yielded a total of 50 publications, of which 42 articles (Table S2) were chosen and used in this review.

### 2.2. The Collected Prophage Gene Clusters Identified in the Vibrio spp. Genomes

Based on the retrieved articles, we collected 149 intact prophage gene clusters identified in the genomes of 82 *Vibrio* isolates, which belonged to seven *Vibrio* species, including *V. alginolyticus*, *Vibrio campbellii*, *V. cholerae*, *Vibrio gazogenes*, *Vibrio nigripulchritudo*, *V. parahaemolyticus*, and *Vibrio penaeicida* (Table S1). The relationships of these prophages were plotted and visualized using the SRplot software [31].

## 3. Results and Discussion

### 3.1. Prophages Contribute to the Virulence of Vibrio spp.

A well-known mechanism of virulence acquisition in *Vibrio* is the integration of CTXΦ phage (10,638 bp, GenBank accession no. NC_015209) into *V. cholerae* [32]. CTXΦ, which commonly exists in the 7th pandemic serogroup O1 and toxigenic serogroup O139 strains, encodes cholera toxin (CT) and toxin-coregulated pilus (TCP) [33]. The CT can lead to severe disruption of intestinal cell function, the watery diarrhea of cholera [34], while TCP is the receptor for entry of CTXΦ into the cell [35]. Another toxin encoded by CTXΦ and found in *Vibrio* prophages is zonula occludens toxin (Zot), the C-terminal domain of which displays enterotoxic activity [36]. This toxin increases the permeability of the surface of marine plankton or the host intestinal cell [37]. Recently, Castillo et al. reported that 28 of 64 *Vibrio* species harbored prophages that encoded the Zot toxin [38]. Accessory cholera enterotoxin (Ace) is another toxin found in *V. cholerae* lysogen that is also responsible for diarrhea in cholera [34]. Most recently, a number of *zot*-like and *ace*-like genes were identified in *Vibrio* phages, such as the VSK (6882 bp, GenBank accession no. NC_003327) in *V. cholerae*, f237 (8784 bp, GenBank accession no. AP000581) and VF33 (7965 bp, GenBank accession no. NC_005948) in *V. parahaemolyticus*, and VALGΦ6 (8529 bp, GenBank accession no. MN719123) in *V. alginolyticus* [39,40,41], which could be biological reservoirs for the emergence of pathogenic clones of *Vibrio* spp.

Phages can escape degradation in the host cell by methylating their own DNA using DNA adenine methyltransferase (DAM) [42]. Therefore, DAM is considered to be one of the important enzymes related to *Vibrio* virulence. Previous research has indicated that DAM likely contributed to the switching between lytic and lysogenic life cycles of the *V. harveyi* Myoviridae-like (VHML) phage (43,198 bp, GenBank accession no. NC_004456) by the methylation and subsequent inhibition of the lytic repressor gene *CI*, similarly to the *rha* antirepressor gene of *Escherichia coli* phage Φ80 (46,150 bp, GenBank accession no. NC_021190) [42]. Some phages are involved in *Vibrio* virulence by up-regulating key virulence-associated genes. For example, the VHML phage could up-regulate hemolysin excretion in *V. harveyi* 642 [43]. Interestingly, *V. harveyi* strains infected with the VHML phage needed fewer nutrients than the uninfected ones, which may help the lysogenized bacteria to be more competitive in nutrient-limited circumstance [43,44]. Recently, Santoriello et al. reported that a type VI secretion system (T6SS) gene cluster Aux3 existed as a mobile and prophage-like element in some environmental *V. cholerae*, excised from and inserted into the host chromosomes via site-specific recombination, but a stable truncated form existed in pandemic strains in that the recombination is reduced, indicating that pandemic *V. cholerae* is inactive in site-specific recombination to retain an interbacterial defense mechanism [45,46]. Most recently, Xu et al. [47] reported a prophage gene cluster in *V. parahaemolyticus* N8-42 genome, which showed sequence similarity to the *Vibrio*_phage_fs2 (8651 bp, NCBI accession number: NC_001956) and encoded type II secretion system (T2SS) and T3SS family protein (GspD) [47]. GspD constructs the outer membrane channel of T2SS, which secretes diverse toxins causing severe diarrhea and cholera [48].

Some prophages are pathogenic to aquatic animal hosts. For instance, the prophage VfO3K6 (8784 bp, GenBank accession no. NC_002362) in *V. parahaemolyticus* SAB6 can cause acute hepatopancreatic necrosis disease (AHPND) in shrimps [49]. Khemayan et al. [50] found that the open read frame (ORF) 058 of a virulence-enhancing siphophage VHS1 (81,509 bp, GenBank accession no. JF713456) in *V. harveyi* likely encoded a toxin. The virulence of *V. harveyi* for *Penaeus monodon* was enhanced by more than 100 fold when it was lysogenized with VHS1 [50]. Recently, Wang et al. [51] isolated 33 pathogenic *Vibrio* spp. strains from aquaculture-grown *Penaeus vannamei* in Guangdong and Jiangsu Provinces, China from 2019 to 2022. They identified 108 prophages in the genomes of these *Vibrio* isolates, including intact prophages (*n* = 40), questionable prophages (*n* = 11), and incomplete prophages (*n* = 57), via the online software PHASTER (https://phaster.ca/) to score an identified DNA sequence region on the basis of the coding sequence count and the presence or absence of phage-associated genes. If its score was above 90, between 70 and 90, or below 70, then the predicted prophage was defined as intact, questionable, or incomplete, respectively. Two of these prophages carried virulence genes, e.g., the T6SS virulence genes in the questionable prophage of *V. parahaemolyticus* A1, and virulence genes *wbfV/wcvB* and *wecA* in the incomplete prophage of *V. alginolyticus* B14 [51]. Most recently, Mesa et al. [52] reported that *V. harveyi* PH1009, isolated from Masbate Island, Philippines, was co-infected with the white spot syndrome virus in *P. monodon*. Two prophage regions were found in the *V. harveyi* PH1009 genome, one of which carried the *zot* and *ace* genes.

### 3.2. Prophages Amplify the Ecological Persistence of Vibrio spp.

Recently, Chen’s research group reported a prophage-like gene cluster in *V. parahaemolyticus* CHN25, which showed high sequence similarity to the *Vibrio* phage martha 12B12 (33,277 bp, GenBank accession no. NC_021070). Twenty-four genes were predicted, encoding phage proteins (*n* = 7, e.g., phage head, tail, and baseplate), regulators (*n* = 8), and hypothetical proteins (*n* = 9). They demonstrated that the unknown genes *VpaChn25_0724*, *VpaChn25_0734*, *VpaChn25_0713*, *VpaChn25_0714*, and *VpaChn25_RS25055* play essential roles in the environmental adaptability of the host [53,54,55]. For example, biofilm formation and cytotoxicity were significantly reduced in the *ΔVpaChn25_0724*, *ΔVpaChn25_0734*, *ΔVpaChn25_0713*, *ΔVpaChn25_0714*, and *ΔVpaChn25_RS25055* mutants, as compared to the wild type *V. parahaemolyticus* CHN25 (*p* < 0.05). Remarkably, knock out of the *VpaChn25_0724* gene caused cell membrane damage and increased cell surface hydrophobicity of *V. parahaemolyticus* CHN25 [53]. The deletion of *VpaChn25_0734* and *VpaChn25_RS25055* genes decreased cell surface hydrophobicity of *V. parahaemolyticus* CHN25; the deletion of the *VpaChn25_0713* gene led to a reduction in internal membrane permeability of *V. parahaemolyticus* CHN25; and the deletion of *VpaChn25_RS25055*, *VpaChn25_0713*, and *VpaChn25_0714* genes resulted in higher cell membrane fluidity of *V. parahaemolyticus* CHN25 [54,55]. Comparative secretomic analysis uncovered a significant increase in extracellular proteins, e.g., OmpW, FlaB/D, and FlaA flagellins, aldehyde-alcohol dehydrogenase (AdhE), phage head morphogenesis protein, and D-lactate dehydrogenase (D-LDH), in the *ΔVpaChn25_0724* mutant, as compared to the wild-type strain (*p* < 0.05). Comparative transcriptomic analysis revealed 12 significantly changed metabolic pathways in the *ΔVpaChn25_0724* mutant, indicating the shunt transport and carbon source usage, and inhibited energy production and membrane biosynthesis (*p* < 0.05). Several notably repressed key regulators in bacterial gene regulatory networks were closely related to phenotypic variations [53]. In the *VpaChn25_0734* gene deletion mutant, the secretion of 30S ribosomal protein S1 (RpsA) and DNA-directed RNA polymerase subunit alpha (RpoA) was significantly increased (*p* < 0.05). Comparative transcriptomic analyses also revealed 13 significantly altered metabolic pathways in the *ΔVpaChn25_0734* mutant, including the repressed carbon source transport and utilization, biofilm formation, and T2SS, linked to the defective phenotypes [54]. Additionally, transcriptomic analysis also revealed 15, 14, and 8 significantly altered metabolic pathways in the absence of *VpaChn25_RS25055*, *VpaChn25_0713*, and *VpaChn25_0714* genes, respectively (*p* < 0.05) [55]. Most recently, Zhao et al. found that the protein encoded by *VpaChn25_RS25055* existed at both poles of the *V. parahaemolyticus* CHN25 cell [55].

Recently, Li et al. [56] also reported an intact prophage-like element Vaf1 (10,004 bp, GenBank accession no. OP297622) in the genome of the emerging marine pathogen *V. alginolyticus* AP-1. Vaf1 significantly promoted biofilm formation, swarming motility, and contact-dependent competition of *V. alginolyticus* AP-1 [56]. The in vivo zebrafish (*Danio rerio*) infection mode experiment evidenced that Vaf1 contributed to the virulence of *V. alginolyticus* AP-1 [56]. Most recently, Chen’s research group also reported five prophage regions in *V. parahaemolyticus* N1-22, N4-46, N8-42, and Q8-15 genomes [47]. For instance, the *V. parahaemolyticus* N8-42 genome carried two prophage regions with sequence identity to the *Vibrio*_phage_K139 (33,106 bp, GenBank accession no. NC_003313) and *Vibrio*_phage_fs2 (8651 bp, GenBank accession no. NC_001956), respectively. The *Vibrio*_phage_K139 homologue also existed in *V. parahaemolyticus* N1-22. It contained 46 genes, encoding phage major structure genes that were the same as those in *V. parahaemolyticus* N8-42, as well as several different accessory genes, implying more recent HGT and genome recombination amongst the *V. parahaemolyticus* isolates. Moreover, the *Pseudomonas*_phage_D3 (56,426 bp, GenBank accession no. NC_002484) homologues were found in *V. parahaemolyticus* N4-46 and Q8-15 genomes, carrying 28 and 29 genes, respectively, indicating possible HGT across different genera of *Pseudomonas* and *Vibrio* [47].

Most recently, Qin et al. [57] also reported six prophage gene clusters that contained 248 predicted genes in *V. cholerae* L1-1, L10-48, and B5-86 genomes, 53.6% of which encoded unknown proteins. For instance, *V. cholerae* L1-1 carried three prophage regions, which displayed sequence identity to the *Escherichia*_phage_lys12581Vzw (62,668 bp, GenBank accession no. NC_049917), *Vibrio*_phage_VHML, and *Vibrio*_phage_VCY_phi (7103 bp, GenBank accession no. NC_016162), respectively. *V. cholerae* L10-48 had two prophage regions, with sequence identity to the *Burkholderia_cenocepacia*_phage_BcepMu (36,748 bp, GenBank accession no. NC_005882) and *Escherichia*_phage_ArgO145 (62,020 bp, GenBank accession no. NC_049918), respectively. One *Escherichia*_converting_phage_Stx2a_F451 (64,900 bp, GenBank accession no. NC_049924) homologue existed in *V. cholerae* B5-86. Notably, the six identified prophages in *V. cholerae* originated from the distinct genera *Burkholderia cenocepacia*, *Escherichia* spp., and *Vibrio* spp., suggesting that the phage transmission likely occurred across these genera boundaries [57].

Recently, Wang et al. [58] investigated culturable bacteria in the gastric cavity of healthy *Galaxea fascicularis*, a scleractinian coral prevalent in coral reef areas of the Indo-Pacific Ocean. They reported that temperate phages are key players in the colonization competition in the coral microbiota. Remarkably, *Vibrio coralliilyticus* outcompeted other coral symbiotic bacteria (e.g., *Endozoicomonas* spp.), via the LodAB-dependent prophage induction, to obtain a competitive advantage over lysogenic competitors when colonizing corals [58]. Coral mucus also serves as a reservoir of bacteriophages targeting *Vibrio* pathogens [59].

### 3.3. Prophages Assist the Superinfection Exclusion in Vibrio spp.

Superinfection exclusion is the ability of bacterial strains carrying temperate phages to resist subsequent infection by the same phage [40]. Superinfection exclusion in the Φ80 in *E. coli* K-12 and HK97 (39,732 bp, GenBank accession no. NC_002167) in *E. coli* 594 have been reported [60,61]. However, the underlying mechanism has not been unveiled yet. Kalatzis et al. [62] reported that the H20 (53,224 bp, GenBank accession no. KY658675)-like phage in *Vibrio anguillarum* A023, T265, BA35, and VaKef strains harbored a repressor gene that genetically and structurally resembled a repressor of the *Escherichia*_phage_λ (48,502 bp, GenBank accession no. NC_001416). It possibly blocked the gene expression of the lytic pathway and conferred repressor-mediated immunity to other H20-like phages in *V. anguillarum* [62]. Additionally, it has also been reported that once the VHS1 phage initiated the infection, *V. harveyi* inhibited and prevented the VHS1 binding receptor from adhering to another phage particle [63].

Pseudolysogens are bacteria that are infected with pseudolysogenic phages that can not became a stable lysogen, e.g., the 493 phage (9300 bp) in *V. cholerae* and the VHS1 in *V. harveyi*. Pseudolysogens are classified into two groups: (1) those that contain a defective phage genome and can resist superinfection but do not produce phage particles; (2) those that tolerate superinfection but do not contain the phage genome [40,64]. Recently, a new pre-CTXΦ prophage was found in *V. cholera* of serogroup O139. The immunity to CTXΦ superinfection triggered by the *rstR* allele of this pre-CTXΦ is phage-specific [65]. Notably, recently, Pant et al. [66] found that the core genome-encoded RecA helped CTXΦ to bypass *V. cholera* immunity to replicate in the host, as a similar prophage existed in the host chromosomes [66].

### 3.4. Prophages Promote the Genome Evolution of Vibrio spp.

**Figure 1:**
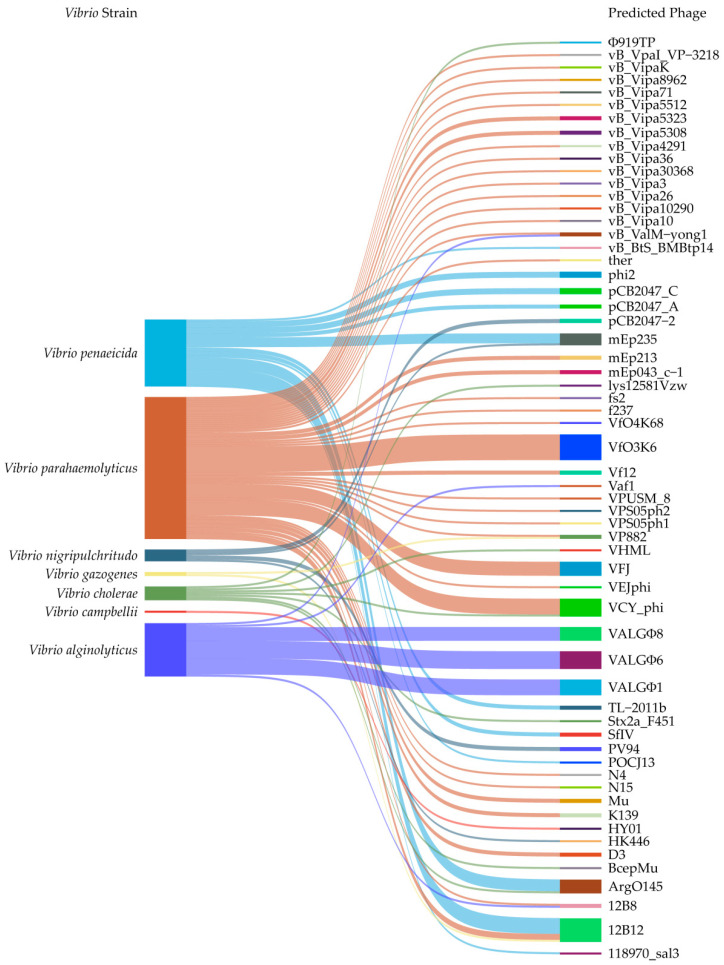
Sankey diagram showing the relationship of the collected 149 predicted prophage gene clusters (Table S1) identified in genomes of 82 *Vibrio* isolates in the most recent 5 years. The diagram was plotted and visualized using the SRplot online platform (https://www.bioinformatics.com.cn/srplot, accessed on 15 December 2024).

As shown in Figure 1, the collected 149 intact prophage gene clusters carrying a number of genes were present in the genomes of 82 *Vibrio* isolates belonging to seven *Vibrio* species. They showed sequence similarity to 60 different phages, promoting the genome evolution of *Vibrio* spp. For instance, the *Vibrio*_phage_henriette_12B8 (107,218 bp, GenBank accession no. NC_021073) was found in *V. alginolyticus* and *V. parahaemolyticus* genomes; the *Escherichia*_phage_ArgO145 (62,020 bp, GenBank accession no. NC_049918) in *V. cholerae* and *V. penaeicida* genomes; the *Vibrio*_phage_VP882 (38,197 bp, GenBank accession no. NC_009016) in *V. gazogenes* and *V. parahaemolyticus* genomes; and the *Enterobacteria*_phage_mEp235 (37,595 bp, GenBank accession no. NC_019708) in *V. nigripulchritudo* and *V. penaeicida* genomes (Figure 1).

Studies have evidenced *Vibrio* spp. undergoing prophage-mediated evolution [67]. For instance, *V. cholerae* of El Tor biotype can replicate the CTXΦ genome and secrete CTXΦ phage particles. Due to space constraints, readers are referred to reviews on the gene organization of CTXΦ [67]. Recently, Ochi et al. [68] analyzed complete genomes of five clinical *V. cholerae* strains isolated in Kolkata, India during the period from 2007 to 2011. They found that recent strains had a changed CTXΦ array and could not replicate the CTXΦ genome. Further analysis revealed the identical *rstA* and intergenic sequence 1 (Ig-1) in all the recent isolates in the altered CTXΦ array [68]. Diarrheal cases caused by non-toxigenic *V. cholerae* have been reported globally [69]. Lineages L3b and L9, featured as *ctxAB*-negative and *tcpA*-positive, pose the most dangerous risk and caused long-term epidemics in different regions in the world [70]. Two waves (2001–2012 and 2013–2018) of cholera were reported in Hangzhou, China, between 2001 and 2018 and were caused by non-toxigenic *V. cholerae* [71]. Hao et al. [71] analyzed 207 genomes of Hangzhou isolates from these two waves together with 1573 publicly available genomes and found that 21% of L3b and L9 isolates had become CT producers, indicating that the gain of complete CTXΦ-carrying *ctxAB* genes led to the transition [71]. Recently, Behera et al. [72] reported *ctxB*, *tcpA*, and *rstR* genes in CTX prophages among *V. cholerae* isolates of serotype O139 (*n* = 59) in Odisha, India from 1999 to 2017 and found three genotypes, 1, 3, and 4, with at least one copy of CTX^Calc^Φ in addition to CTX^ET^ and CTX^Cl^ in the *V. cholerae* isolates [72]. Additionally, Thong et al. [73] investigated *V. cholerae* strains of the El Tor biotype (*n* = 45) related to outbreaks and sporadic cases in Malaysia between 1991 and 2011. They found that the outbreak strains isolated in 1991 carried the El Tor CT gene (*ctxB3*), whereas sporadic strains from 2004 to 2011 contained the classical *ctxB1* gene. Four different CTXΦ arrays existed in the El Tor variants, one of which co-existed with El Tor strains in the 2009 outbreak in Terengganu, Malaysia [73].

The epidemiology of *V. parahaemolyticus* remarkably changed in America following the Pacific-native lineage called sequence type (ST) 36 in the Atlantic [74]. Most recently, Foxall et al. reported that the major genomic differentiation and competitive success of ST36 included the loss of an inovirus prophage that had been maintained for decades in the endemic north Pacific population [41]. Broader surveys indicated that inoviruses are common and active among the global population of *V. parahaemolyticus*, and inovirus replacements are infrequent, such as in ST36; however, it is worth noting that they exist in pathogenic lineages that dispersed [41].

Studies have shown that HGT events between different *Vibrio* spp. (e.g., *Vibrio fischeri* and *V. cholerae*) were promoted by mobile genetic elements (MGEs). For example, a CTXΦ phage-like gene cluster was identified in *V. fischeri* ES114 genome, leading to the virulence genes in CTXΦ from *V. cholerae* transferred to *V*. *mimicus* [75]. Wang et al. [76] identified the two prophages VPS05ph1 (33,915 bp) and VPS05ph2 (39,156 bp) in *V. parahaemolyticus* S05, showing 38.3% sequence similarity to each other. The majority of the predicted genes (18/24) in VPS05ph1 were similar to the *Vibrio* phage VPUSM 8 (34,145 bp, GenBank accession no. NC_022747), whereas most genes (19/32) in VPS05ph2 were similar to the *Aeromonas* phage phiO18P (33,985 bp, GenBank accession no. NC_009542) but showing sequence similarity of only 18.7% [76]. Most recently, Soto et al. [77] searched 4619 *Vibrio* genomes from 127 species for prophages carrying the *zot* gene. They found 2030 potential prophages with *zot*-like genes in 43 *Vibrio* species. Some prophages, such as CTXΦ or Vf33, were associated with specific species, whereas prophages VCY_phi and VfO3K6 were found in 28 and 20 *Vibrio* species, respectively [77]. *V. campbellii* is an emerging pathogen in aquaculture that can cause luminescent vibriosis in farmed shrimp. Nuidate et al. [78] determined the genome sequence of prophage HY01 (41,772 bp, GenBank accession no. MT366580) in *V. campbellii*, which was distantly associated with the *Vibrio* phage Va_PF430-3_p42 (51,907 bp, GenBank accession no. MK672805) in *V. anguillarum*. A total of 60 ORFs of HY01 were predicted, of which 31 had known biological functions. Sequence analysis of clustered regularly interspaced short palindromic repeats (CRISPR) spacers showed two matching sequences between the HY01 genome and viral spacer sequence of *Vibrio* spp. [78].

The lysogenic phage CTXΦ harbors the precursor genome without the *ctxAB*, named pre-CTXΦ. Multiple types of the pre-CTXΦ have been found in toxigenic and non-toxigenic *V. cholerae* strains, based on the transcriptional regulator gene *rstR* alleles in CTXΦ/pre-CTXΦ [79]. A pre-CTXΦ genome type carrying a new *rstR* allele, pre-CTX^ZHJ^Φ, was identified. This pre-CTX^ZHJ^Φ inserted in the small chromosome of *V. cholerae* and coexisted with a typical CTX^ET^Φ prophage in the large chromosome. RstR^ZHJ^ was able to bind to the intergenic sequence 2 (Ig-2) in the *rstAB* promotor in the pre-CTX^ZHJ^Φ genome and inhibited its own *rstAB* gene expression but did not inhibit this gene in CTX^ET^Φ and CTX^class^Φ. These results suggested that *V. cholerae* isolates harboring the pre-CTX^ZHJ^Φ cannot escape from the infection of CTXΦs, thus potentially converting into toxigenic strains [65].

Recently, Garin-Fernandez et al. reported the genome structure of the inducible phage vB_VpaI_VP-3218 (11,082 bp, GenBank accession no. LR595856), a novel filamentous phage in *V*. *parahaemolyticus* VN-3218 isolated from the North Sea [80]. This phage can integrate into the host chromosome along with other *Vibrio* host genomes from the environment. These results suggested that a number of prophage-mediated HGT events occurred during the evolution of *Vibrio* spp.

### 3.5. Regulation of Prophages in Vibrio spp.

Studies have shown that CI repressor concentration and cell density regulate prophage induction in *V. cholerae* and *V. anguillarum*, respectively [81,82,83,84]. For instance, the phage Φ919TP (33,133 bp, GenBank accession no. KU504502) in *V. cholerae* was a λ-like phage, harboring a CI repressor that governed the lysis/lysogeny switch in the host cell [81,82]. When the CI repressor concentration was high, it repressed the development of the lytic life cycle of the Φ919TP [82]. Recently, Li et al. [83] analyzed a Φ919TP-deleted variant of *V. cholerae* and its interaction with a modified lytic variant of the induced prophage (Φ919TP *cI*^−^). Comparison of wild-type and Φ919TP *cI^−^*-resistant mutant genomes revealed that the Φ919TP *cI*^−^ selected for phage-resistant mutants in the key steps of lipopolysaccharide (LPS) O-antigen biosynthesis were targeted, resulting in a single-base-pair deletion in the *gmd* gene. The *gmd*-mediated O-antigen defect led to pleiotropic phenotypes, e.g., cell auto-aggregation and decreased swarming motility [83]. Tan et al. [84] investigated the effect of quorum sensing (QS) on the interactions between *V. anguillarum* 90-11-287 and its H20-like prophage p41 (54,432 bp, GenBank accession no. MK672799). They found that the induction of the H20-like prophage was governed by the QS state of the host, and phage particles increased by eight fold per cell in high-cell-density (HCD) cultures of the QS-deficient *ΔvanT* mutant. Compared to prophage-free strain, H20-like prophage enhanced biofilm formation in low-cell-density (LCD) cultures. Conversely, the HCD state was linked to the decreased prophage induction, the enhanced proteolytic activity, and the inhibited biofilm formation. The intertwined regulation of phage-host interactions and biofilm formation suggested that the increased lysogeny at HCD was not only a successful strategy for phages to utilize in bacterial hosts, but also was a host strategy evolved to govern the lysis–lysogeny switch to benefit the host survival [84,85,86].

Additionally, Xu et al. [87] recently evaluated the potential application of prophages in identifying *Vibrio* species and strains. They found that prophages in *Vibrio* strains were highly specific at the strain and species level, implying that prophages could be potentially applied in microbial species, sub-species, and strain-level identification [87].

## 4. Conclusions

The genus *Vibrio* includes at least 152 species, 12 of which have been implicated in human severe cholera and vibriosis diseases. These bacteria are frequently detected in aquatic products worldwide, posing a huge danger to food safety and public health. In this review, we searched and collected 149 newly discovered intact prophages located in the genomes of 82 *Vibrio* spp. in the most recent 5 years. Based on these data, we deciphered the evolutionary relationship between prophages and *Vibrio* species and highlighted the impact of prophages on the bacterial pathogenicity, environmental fitness, and genome evolution.

Prophages serve as biological reservoirs for the emergence of pandemic, epidemic, or pathogenic clones of *Vibrio* spp., particularly through the transmission of toxin-encoding genes (e.g., *ct*, *tcp*, *zot*, and *ace*, as well as T6SS, T3SS, and T2SS-related virulence factors) across genera or species boundaries. However, molecular mechanisms underlying the transmission need to be further investigated. Recent studies also revealed that some prophages (e.g., VHML) carried genes encoding enzymes (e.g., DAM) switching between lytic and lysogenic life cycles to avoid degradation in the host cell; some contributed to *Vibrio* virulence by up-regulating key virulence-associated genes; and some were pathogenic to aquatic animal hosts as well.

Prophages amplify the ecological persistence of *Vibrio* spp. Recently, the prophages identified in *Vibrio* spp. genomes were found to carry a number of genes, endowing the bacteria with additional biological functions (e.g., resistance, competition, and substance metabolism) besides virulence. Notably, in *V. parahaemolyticus*, some prophage-related genes (e.g., *VpaChn25_0724*, *VpaChn25_0734*, *VpaChn25_0713*, *VpaChn25_0714*, and *VpaChn25_RS25055*) have been demonstrated to play essential roles in the bacterial biofilm formation, cell structure integrity, low-temperature survival, and cytotoxicity to the host. In *V. alginolyticus*, the presence of Vaf1 has been evidenced to significantly increase bacterial biofilm formation, swarming motility, and contact-dependent competition. In *V. coralliilyticus*, LodAB-dependent prophage induction contributed to a bacterial competitive advantage over lysogenic competitors when colonizing corals.

Prophages promote the genome evolution of *Vibrio* spp. The collected 149 intact prophages carrying a number of genes were present in the genomes of 82 *Vibrio* isolates belonging to seven *Vibrio* species. They showed sequence similarity to 60 different phages, substantially promoting the genome evolution of *Vibrio* spp. Remarkably, recent studies have evidenced *Vibrio* spp. undergoing prophage-mediated evolution, such as the CTXΦ in clinical *V. cholerae* strains and the filamentous prophage in *V. parahaemolyticus* pathogenic lineages. Moreover, a number of prophage-mediated HGT events likely occurred between different *Vibrio* spp. (e.g., *Vibrio fischeri* and *V. cholerae*) or across genera boundaries (e.g., *Burkholderia*, *Escherichia*, and *Vibrio*). On the other hand, prophages assisted superinfection exclusion in *Vibrio* spp., leading to the integration into the host genomes, e.g., the H20-like phage in *V. anguillarum* and VHS1 in *V. harveyi.* In addition, the intertwined regulation of phage–host interactions, such as between Φ919TP and *V. cholerae* and between H20-like prophage p41 and *V. anguillarum*, have recently been reported as well.

Overall, this review facilitates a better understanding of the prophage-promoting evolution of *Vibrio* spp. and their implications for food safety and public health.

## Data Availability

No new data were created or analyzed in this study. Data sharing is not applicable to this article.

## Supplementary Materials

The following supporting information can be downloaded at: https://www.mdpi.com/article/10.3390/foods14030403/s1, Table S1. The intact prophage gene clusters identified in *Vibrio* spp. in the most recent 5 years [80,88,89,90,91,92,93]; Table S2. Information on the 42 articles selected in this review.

## References

[1] Lukjancenko O., Ussery D.W. (2014). Vibrio chromosome-specifific families. Front. Microbiol..

[2] Serratore P., Bignami G., Ostanello F., Lorito L. (2021). Hazard identification related to the presence of *Vibrio* spp., biogenic amines, and indole-producing bacteria in a non-filter feeding marine gastropod (*Tritia mutabilis*) commercialized on the Italian market. Foods.

[3] Zeidler C., Szott V., Alter T., Huehn-Lindenbein S., Fleischmann S. (2024). Prevalence of *Vibrio* spp. in seafood from German supermarkets and fish markets. Foods.

[4] Thompson F.L., Iida T., Swings J. (2004). Biodiversity of Vibrios. Microbiol. Mol. Biol. Rev..

[5] Baker-Austin C., Oliver J.D., Alam M., Ali A., Waldor M.K., Qadri F., Martinez-Urtaza J. (2018). *Vibrio* spp. infections. Nat. Rev. Dis. Primer.

[6] Grimes D.J. (2020). The Vibrios: Scavengers, symbionts, and pathogens from the sea. Microb. Ecol..

[7] Hsueh B.Y., Waters C.M. (2019). Combating cholera. F1000Research.

[8] Campos L.C., Zahner V., Avelar K.E.S., Alves R.M., Pereira D.S.G., Vital B.J.M., Freitas F.S., Salles C.A., Karaolis D.K.R. (2004). Genetic diversity and antibiotic resistance of clinical and environmental suggests that many serogroups are reservoirs of resistance. Epidemiol. Infect..

[9] Chaguza C., Chibwe I., Chaima D., Musicha P., Ndeketa L., Kasambara W., Mhango C., Mseka U.L., Bitilinyu-Bangoh J., Mvula B. (2024). Genomic insights into the 2022–2023 *Vibrio cholerae* outbreak in Malawi. Nat. Commun..

[10] Chen D., Li X., Ni L., Xu D., Xu Y., Ding Y., Xie L., Chen L. (2021). First experimental evidence for the presence of potentially toxic *Vibrio cholerae* in snails, and virulence, cross-resistance and genetic diversity of the bacterium in 36 species of aquatic food animals. Antibiotics.

[11] Yan L., Jin Y., Zhang B., Xu Y., Peng X., Qin S., Chen L. (2022). Diverse aquatic animal matrices play a key role in survival and potential virulence of non-O1/O139 *Vibrio cholerae* isolates. Front. Microbiol..

[12] Ceccarelli D., Hasan N.A., Huq A., Colwell R.R. (2013). Distribution and dynamics of epidemic and pandemic *Vibrio parahaemolyticus* virulence factors. Front. Cell. Infect. Microbiol..

[13] Meza G., Majrshi H., Tiong H.K. (2022). Recovery of pasteurization-resistant *Vibrio parahaemolyticus* from seafoods using a modified, two-step enrichment. Foods.

[14] Wu K., Zou D., Long Y., Xue L., Shuai S., Tian F., Li M., Fan G., Zheng Y., Sun X. (2024). Contamination of *Vibrio parahaemolyticus* in crayfish for sale. Front Microbiol..

[15] Hughes M.J., Flaherty E., Lee N., Robbins A., Weller D.L. (2024). Severe *Vibrio vulnificus* infections during heat waves—Three eastern U.S. States, July–August 2023. MMWR Morb. Mortal. Wkly. Rep..

[16] Yamazaki K., Kashimoto T., Kado T., Yoshioka K., Ueno S. (2022). Increased vascular permeability due to spread and invasion of *Vibrio vulnificus* in the wound infection exacerbates potentially fatal necrotizing disease. Front. Microbiol..

[17] Liu W., Zhang G., Xu D., Ye J., Lu Y. (2023). A novel RAA combined test strip method based on dual gene targets for pathogenic *Vibrio vulnificus* in aquatic products. Foods.

[18] Jacobs Slifka K.M., Newton A.E., Mahon B.E. (2017). *Vibrio alginolyticus* infections in the USA, 1988-2012. Epidemiol. Infect..

[19] Abdelsalam M., Attia M.M., Marzouk M.S., Korany R.M.S., Elgendy M.Y., Soliman A.W., Prince A., Hamada A.H. (2024). Investigating dynamics, etiology, pathology, and therapeutic interventions of *Caligus clemensi* and *Vibrio alginolyticus* co-infection in farmed marine fish. Sci. Rep..

[20] Sun Y., Yan Y., Yan S., Li F., Li Y., Yan L., Yang D., Peng Z., Yang B., Sun J. (2024). Prevalence, antibiotic susceptibility, and genomic analysis of *Vibrio alginolyticus* isolated from seafood and freshwater products in China. Front Microbiol..

[21] Morgado M.E., Brumfield K.D., Mitchell C., Boyle M.M., Colwell R.R., Sapkota A.R. (2024). Increased incidence of vibriosis in Maryland, U.S.A., 2006–2019. Environ. Res..

[22] Fortier L.C., Sekulovic O. (2013). Importance of prophages to evolution and virulence of bacterial pathogens. Virulence.

[23] Shkoporov A.N., Turkington C.J., Hill C. (2022). Mutualistic interplay between bacteriophages and bacteria in the human gut. Nat. Rev. Microbiol..

[24] Wendling C.C., Refardt D., Hall A.R. (2021). Fitness benefits to bacteria of carrying prophages and prophage-encoded antibiotic-resistance genes peak in different environments. Evolution.

[25] Dedrick R.M., Jacobs-Sera D., Bustamante C.A.G., Garlena R.A., Mavrich T.N., Pope W.H., Reyes J.C.C., Russell D.A., Adair T., Alvey R. (2017). Prophage-mediated defence against viral attack and viral counter-defence. Nat. Microbiol..

[26] Garin-Fernandez A., Wichels A. (2020). Looking for the hidden: Characterization of lysogenic phages in potential pathogenic *Vibrio* species from the North Sea. Mar. Genom..

[27] Steensen K., Séneca J., Bartlau N., Yu X.A., Hussain F.A., Polz M.F. (2024). Tailless and filamentous prophages are predominant in marine Vibrio. ISME J..

[28] Castillo D., Pérez-Reytor D., Plaza N., Ramírez-Araya S., Blondel C.J., Corsini G., Bastías R., Loyola D.E., Jaña V., Pavez L. (2018). Exploring the genomic traits of non-toxigenic *Vibrio parahaemolyticus* strains isolated in Southern Chile. Front. Microbiol..

[29] Kobakhidze S., Koulouris S., Kakabadze N., Kotetishvili M. (2024). Genetic recombination-mediated evolutionary interactions between phages of potential industrial importance and prophages of their hosts within or across the domains of *Escherichia*, *Listeria*, *Salmonella*, *Campylobacter*, and *Staphylococcus*. BMC Microbiol..

[30] Molina-Quiroz R.C., Dalia T.N., Camilli A., Dalia A.B., Silva-Valenzuela C.A. (2020). Prophage-dependent neighbor predation fosters horizontal gene transfer by natural transformation. mSphere.

[31] Tang D., Chen M., Huang X., Zhang G., Zeng L., Zhang G., Wu S., Wang Y. (2023). SRplot: A free online platform for data visualization and graphing. PLoS ONE.

[32] Davis B.M., Moyer K.E., Boyd E.F., Waldor M.K. (2000). CTX prophages in classical biotype *Vibrio cholerae*: Functional phage genes but dysfunctional phage genomes. J. Bacteriol..

[33] Biswas Q., Purohit A., Kumar A., Rakshit D., Maiti D., Das B., Bhadra R.K. (2022). Genetic and mutational analysis of virulence traits and their modulation in an environmental toxigenic *Vibrio cholerae* non-O1/non-O139 strain, VCE232. Microbiology.

[34] Miller C.E., Majewski J., Faller R., Satija S., Kuhl T.L. (2004). Cholera toxin assault on lipid monolayers containing ganglioside GM1. Biophys. J..

[35] Krukonis E.S., DiRita V.J. (2003). From motility to virulence: Sensing and responding to environmental signals in *Vibrio cholerae*. Curr. Opin. Microbiol..

[36] Pérez-Reytor D., Jaña V., Pavez L., Navarrete P., García K. (2018). Accessory toxins of *Vibrio* pathogens and their role in epithelial disruption during infection. Front. Microbiol..

[37] Marinaro M., Fasano A., Magistris M.T.D. (2003). Zonula occludens toxin Acts as an adjuvant through different mucosal routes and induces protective immune responses. Infect. Immun..

[38] Castillo D., Kauffman K., Hussain F., Kalatzis P., Rørbo N., Polz M.F., Middelboe M. (2018). Widespread distribution of prophage-encoded virulence factors in marine *Vibrio* communities. Sci. Rep..

[39] Chibani C.M., Hertel R., Hoppert M., Liesegang H., Wendling C.C. (2020). Closely related *Vibrio alginolyticus* strains encode an identical repertoire of caudovirales-like regions and filamentous phages. Viruses.

[40] Nawel Z., Rima O., Amira B. (2022). An overview on *Vibrio* temperate phages: Integration mechanisms, pathogenicity, and lysogeny regulation. Microb. Pathog..

[41] Foxall R.L., Means J., Marcinkiewicz A.L., Schillaci C., DeRosia-Banick K., Xu F., Hall J.A., Jones S.H., Cooper V.S., Whistler C.A. (2024). Inoviridae prophage and bacterial host dynamics during diversification, succession, and Atlantic invasion of Pacific-native *Vibrio parahaemolyticus*. mBio.

[42] Bochow S., Elliman J., Owens L. (2012). Bacteriophage adenine methyltransferase: A life cycle regulator? Modelled using *Vibrio harveyi* myovirus like. J. Appl. Microbiol..

[43] Munro J., Oakey J., Bromage E., Owens L. (2003). Experimental bacteriophage-mediated virulence in strains of *Vibrio harveyi*. Dis. Aquat. Organ..

[44] Paul J.H. (2008). Prophages in marine bacteria: Dangerous molecular time bombs or the key to survival in the seas?. ISME J..

[45] Santoriello F.J., Michel L., Unterweger D., Pukatzki S. (2020). Pandemic *Vibrio cholerae* shuts down site-specific recombination to retain an interbacterial defence mechanism. Nat. Commun..

[46] Santoriello F.J., Pukatzki S. (2021). When the pandemic opts for the lockdown: Secretion system evolution in the cholera bacterium. Microb. Cell Graz Austria.

[47] Xu D., Peng X., Xie L., Chen L. (2022). Survival and genome diversity of *Vibrio parahaemolyticus* isolated from edible aquatic animals. Diversity.

[48] Yu Z., Wu Y., Chen M., Huo T., Zheng W., Ludtke S.J., Shi X., Wang Z. (2023). Membrane translocation process revealed by in situ structures of type II secretion system secretins. Nat. Commun..

[49] Yu L.H., Teh C.S.J., Yap K.P., Ung E.H., Thong K.L. (2020). Comparative genomic provides insight into the virulence and genetic diversity of *Vibrio parahaemolyticus* associated with shrimp acute hepatopancreatic necrosis disease. Infect. Genet. Evol..

[50] Khemayan K., Prachumwat A., Sonthayanon B., Intaraprasong A., Sriurairatana S., Flegel T.W. (2012). Complete genome sequence of virulence-enhancing siphophage VHS1 from *Vibrio harveyi*. Appl. Environ. Microbiol..

[51] Wang Z., Wang H., Chen D., Li Y. (2024). Genomic characterization and comparative genomic analysis of pathogenic *Vibrio* isolated from aquaculture-grown white-leg shrimp (*Penaeus vannamei*) in Guangdong and Jiangsu, China. Aquaculture.

[52] Mesa C.A.D., Mendoza R.M., Penir S.M.U., de la Peña L.D., Amar E.C., Saloma C.P. (2023). Genomic analysis of *Vibrio harveyi* strain PH1009, a potential multi-drug resistant pathogen due to acquisition of toxin genes. Heliyon.

[53] Yang L., Wang Y., Yu P., Ren S., Zhu Z., Jin Y., Yan J., Peng X., Chen L. (2020). Prophage-related gene *VpaChn25_0724* contributes to cell membrane integrity and growth of *Vibrio parahaemolyticus* CHN25. Front. Cell. Infect. Microbiol..

[54] Xu Y., Yang L., Wang Y., Zhu Z., Yan J., Qin S., Chen L. (2022). Prophage-encoded gene *VpaChn25_0734* amplifies ecological persistence of *Vibrio parahaemolyticus* CHN25. Curr. Genet..

[55] Zhao H., Xu Y., Yang L., Wang Y., Li M., Chen L. (2024). Biological function of prophage-related gene cluster *ΔVpaChn25*_*RS25055*~*ΔVpaChn25*_*0714* of *Vibrio parahaemolyticus* CHN25. Int. J. Mol. Sci..

[56] Li X., Wang X., Li R., Zhang W., Wang L., Yan B., Zhu T., Xu Y., Tan D. (2023). Characterization of a filamentous Phage, Vaf1, from *Vibrio alginolyticus* AP-1. Appl. Environ. Microbiol..

[57] Qin X., Yang L., Xu Y., Xie L., Wang Y., Chen L. (2024). Growth and genome features of non-O1/O139 *Vibrio cholerae* isolated from three species of common freshwater fish. Diversity.

[58] Wang W., Tang K., Wang P., Zeng Z., Xu T., Zhan W., Liu T., Wang Y., Wang X. (2022). The coral pathogen *Vibrio coralliilyticus* kills non-pathogenic holobiont competitors by triggering prophage induction. Nat. Ecol. Evol..

[59] Rubio-Portillo E., Robertson S., Antón J. (2024). Coral mucus as a reservoir of bacteriophages targeting *Vibrio* pathogens. ISME J..

[60] Lewis J.M., Janda K.E., Kotter D.B., Grose J.H., McCleary W.R. (2023). Characterization of the attachment of three new coliphages onto the ferrichrome transporter FhuA. J. Virol..

[61] Cumby N., Reimer K., Mengin-Lecreulx D., Davidson A.R., Maxwell K.L. (2015). The phage tail tape measure protein, an inner membrane protein and a periplasmic chaperone play connected roles in the genome injection process of *E. coli* phage HK97. Mol. Microbiol..

[62] Kalatzis P.G., Rørbo N.I., Castillo D., Mauritzen J.J., Jørgensen J., Kokkari C., Zhang F., Katharios P., Middelboe M. (2017). Stumbling across the same phage: Comparative genomics of widespread temperate phages infecting the fish pathogen *Vibrio anguillarum*. Viruses.

[63] Stalin N., Srinivasan P. (2017). Efficacy of potential phage cocktails against *Vibrio harveyi* and closely related *Vibrio* species isolated from shrimp aquaculture environment in the south east coast of India. Vet. Microbiol..

[64] Williamson S.J., McLaughlin M.R., Paul J.H. (2001). Interaction of the ΦHSIC virus with its host: Lysogeny or pseudolysogeny?. Appl. Environ. Microbiol..

[65] Li X., Zhao L., Gao H., Chen L., Fan F., Li Z., Fan Y., Li J., Liang W., Pang B. (2020). A novel pre-CTX prophage in the *Vibrio cholerae* serogroup O139 strain. Infect. Genet. Evol..

[66] Pant A., Bag S., Saha B., Verma J., Kumar P., Banerjee S., Kumar B., Kumar Y., Desigamani A., Maiti S. (2020). Molecular insights into the genome dynamics and interactions between core and acquired genomes of *Vibrio cholerae*. Proc. Natl. Acad. Sci. USA.

[67] McLeod S.M., Kimsey H.H., Davis B.M., Waldor M.K. (2005). CTXφ and *Vibrio cholerae*: Exploring a newly recognized type of phage–host cell relationship. Mol. Microbiol..

[68] Ochi K., Mizuno T., Samanta P., Mukhopadhyay A.K., Miyoshi S., Imamura D. (2021). Recent *Vibrio cholerae* O1 epidemic strains are unable to replicate CTXΦ prophage genome. mSphere.

[69] Anderson A.M.L., Varkey J.B., Petti C.A., Liddle R.A., Frothingham R., Woods C.W. (2004). Non-o1 *Vibrio cholerae* septicemia: Case report, discussion of literature, and relevance to bioterrorism. Diagn. Microbiol. Infect. Dis..

[70] Wang H., Yang C., Sun Z., Zheng W., Zhang W., Yu H., Wu Y., Didelot X., Yang R., Pan J. (2020). Genomic epidemiology of *Vibrio cholerae* reveals the regional and global spread of two epidemic non-toxigenic lineages. PLoS Negl. Trop. Dis..

[71] Hao T., Zheng W., Wu Y., Yu H., Qian X., Yang C., Zheng Z., Zhang X., Guo Y., Cui M. (2023). Population genomics implies potential public health risk of two non-toxigenic *Vibrio cholerae* lineages. Infect. Genet. Evol..

[72] Behera D.R., Nayak A.K., Nayak S.R., Nayak D., Swain S., Maharana P.K., Biswal B., Pany S., Pati S., Pal B.B. (2022). Genomic diversities of *ctxB*, *tcpA* and *rstR* alleles of *Vibrio cholerae* O139 strains isolated from Odisha, India. Environ. Microbiol. Rep..

[73] Thong K.L., Tham K.B.L., Ngoi S.T., Tan S.C., Wan Yussof W.N., Ahmad Hanapi R., Mohamad N., Teh C.S.J. (2022). Molecular characterization of *Vibrio cholerae* O1 El Tor strains in Malaysia revealed genetically diverse variant lineages. Transbound. Emerg. Dis..

[74] Abanto M., Gavilan R.G., Baker-Austin C., Gonzalez-Escalona N., Martinez-Urtaza J. (2020). Global expansion of Pacific Northwest *Vibrio parahaemolyticus* sequence type 36. Emerg. Infect. Dis..

[75] Boyd E.F., Moyer K.E., Shi L., Waldor M.K. (2000). Infectious CTXΦ; and the *Vibrio* pathogenicity island prophage in *Vibrio mimicus*: Evidence for recent horizontal transfer between *V. mimicus* and *V. cholerae*. Infect. Immun..

[76] Wang H., Xie G., Huang J. (2024). Genome-based characterization of a novel prophage of *Vibrio parahaemolyticus*, VPS05ph1, a novel member of Peduoviridae. Virology.

[77] Soto E., Alegría M., Sepúlveda F., García K., Higuera G., Castillo D., Fontúrbel F., Bastías R. (2024). Prophages carrying Zot toxins on different *Vibrio* genomes: A comprehensive assessment using multilayer networks. Environ. Microbiol..

[78] Nuidate T., Kuaphiriyakul A., Surachat K., Mittraparp-Arthorn P. (2021). Induction and genome analysis of HY01, a newly reported prophage from an emerging shrimp pathogen *Vibrio campbellii*. Microorganisms.

[79] Maiti D., Das B., Saha A., Nandy R.K., Nair G.B., Bhadra R.K. (2006). Genetic organization of pre-CTX and CTX prophages in the genome of an environmental *Vibrio cholerae* non-O1, non-O139 strain. Microbiology.

[80] Garin-Fernandez A., Glöckner F.O., Wichels A. (2020). Genomic characterization of filamentous phage vB_VpaI_VP-3218, an inducible prophage of *Vibrio parahaemolyticus*. Mar. Genom..

[81] Shen X., Zhang J., Xu J., Du P., Pang B., Li J., Kan B. (2016). The resistance of *Vibrio cholerae* O1 El Tor strains to the typing phage 919TP, a member of K139 phage family. Front. Microbiol..

[82] Oppenheim A.B., Kobiler O., Stavans J., Court D.L., Adhya S. (2005). Switches in bacteriophage Lambda development. Annu. Rev. Genet..

[83] Li N., Zeng Y., Hu B., Zhu T., Svenningsen S.L., Middelboe M., Tan D. (2021). Interactions between the prophage 919TP and its *Vibrio cholerae* host: Implications of *gmd* mutation for phage resistance, cell auto-aggregation, and motility. Viruses.

[84] Tan D., Hansen M.F., de Carvalho L.N., Røder H.L., Burmølle M., Middelboe M., Lo Svenningsen S. (2020). High cell densities favor lysogeny: Induction of an H20 prophage is repressed by quorum sensing and enhances biofilm formation in *Vibrio anguillarum*. ISME J..

[85] Castillo D., Alvise P.D., Xu R., Zhang F., Middelboe M., Gram L. (2017). Comparative genome analyses of *Vibrio anguillarum* strains reveal a link with pathogenicity traits. mSystems.

[86] Castillo D., Andersen N., Kalatzis P.G., Middelboe M. (2019). Large phenotypic and genetic diversity of prophages induced from the fish pthogen *Vibrio anguillarum*. Viruses.

[87] Xu M., Xu M., Tu Q. (2021). Comparative evaluation of Vibrio delineation methodologies in post-genomic era. Environ. Microbiol. Rep..

[88] Qin W., Li D., Xu L., Lin W., Tong Y. (2021). Complete genome analysis of an active prophage of *Vibrio alginolyticus*. Arch. Virol..

[89] Baby B., Vijay D., Philip P.S., Alnuaimi A.A., Almansoori H.M., Areidat S.O., Khan G., Vijayan R., Akhtar M.K. (2023). Complete genome sequence of *Vibrio gazogenes* PB1: An estuarine bacterium capable of producing prodigiosin from starch or cellulose. Front. Mar. Sci..

[90] Rathnapala J.M.S.N., Ragab W., Kawato S., Furukawa M., Nozaki R., Kondo H., Hirono I. (2023). Genomic characterization and identification of virulence-related genes in *Vibrio nigripulchritudo* isolated from white leg shrimp *Penaeus vannamei*. J. Fish. Dis..

[91] Yang L., Yu P., Wang J., Zhao T., Zhao Y., Pan Y., Chen L. (2024). Genomic and transcriptomic analyses reveal multiple strategies for *Vibrio parahaemolyticus* to tolerate sub-lethal concentrations of three antibiotics. Foods.

[92] Ragab W., Kawato S., Nozaki R., Kondo H., Hirono I. (2022). Comparative genome analyses of five *Vibrio penaeicida* strains provide insights into their virulence-related factors. Microb. Genom..

[93] Zago V., Veschetti L., Patuzzo C., Malerba G., Lleo M.M. (2020). Resistome, mobilome and virulome analysis of *Shewanella algae* and *Vibrio* spp. strains isolated in Italian aquaculture centers. Microorganisms.

