# Supplementary material for: Recent Discovery of Diverse Prophages Located in Genomes of Vibrio spp. and Their Implications for Bacterial Pathogenicity, Environmental Fitness, Genome Evolution, Food Safety, and Public Health
Source: Foods. 2025 Jan 26;14(3):403. doi: 10.3390/foods14030403 (PMC11817191; doi:10.3390/foods14030403)
Supplement: Supplementary file 1 [file foods-14-00403-s001.zip › foods-3387798-supplementary.pdf]

Table S1. The intact prophage gene clusters identified in *Vibrio* spp. in the most recent five years.

Vibrio Strain	Predicted Phage	Phage Size (bp)	Accession No.	Reference
V. alginolyticus AP-1	*Vibrio*_phage_Vaf1	10,004	OP297622	[56]
V. alginolyticus B14	*Vibrio*_phage_henriette_12B8	107,218	NC_021073	[51]
V. alginolyticus MCCC 1K04633	*Vibrio*_phage_vB_ValM-yong1	33,851	NC_049477	[88]
V. alginolyticus K01M1	*Vibrio*_phage_VALGΦ6	8,529	MN719123	[39]
	*Vibrio*_prophage_VALGΦ1	—	CP017889 (1386230-1416561)	
V. alginolyticus K04M1	*Vibrio*_phage_VALGΦ6	8,529	MN719123	[39]
	*Vibrio*_prophage_VALGΦ1	—	CP017891 (1389760-1420091)	
V. alginolyticus K04M3	*Vibrio*_phage_VALGΦ6	8,529	MN719123	[39]
	*Vibrio*_phage_VALGΦ8	7,311	MN690600	
	*Vibrio*_prophage_VALGΦ1	—	CP017896 (1367485-1397816)	
V. alginolyticus K04M5	*Vibrio*_phage_VALGΦ6	8,529	MN719123	[39]
	*Vibrio*_phage_VALGΦ8	7,311	MN690600	
	*Vibrio*_prophage_VALGΦ1	—	CP017899 (1367485-1397816)	
V. alginolyticus K05K4	*Vibrio*_phage_VALGΦ6	8,529	MN719123	[39]
	*Vibrio*_phage_VALGΦ8	7,311	MN690600	
	*Vibrio*_prophage_VALGΦ1	—	CP017902 (1383927-1414258)	
V. alginolyticus K06K5	*Vibrio*_phage_VALGΦ6	8,529	MN719123	[39]
	*Vibrio*_prophage_VALGΦ1	—	CP017907 (1389236-1419567)	
V. alginolyticus K08M3	*Vibrio*_phage_VALGΦ6	8,529	MN719123	[39]
	*Vibrio*_prophage_VALGΦ1	—	CP017913 (1384771-1415102)	
V. alginolyticus K10K4	*Vibrio*_phage_VALGΦ6	8,529	MN719123	[39]
	*Vibrio*_phage_VALGΦ8	7,311	MN690600	
	*Vibrio*_prophage_VALGΦ1	—	CP017911 (1367484-1397815)	
V. campbellii HY01	*Vibrio*_phage_HY01	41,772	MT366580	[78]
V. cholerae L1-1	*Escherichia*_phage_lys12581Vzw	62,668	NC_049917	[57]

	*Vibrio*_phage_VHML	43,198	NC_004456	
	*Vibrio*_phage_VCY_phi	7,103	NC_016162	
V. cholerae L10-48	*Burkholderia_cenocepacia*_phage_BcepMu	36,748	NC_005882	[57]
	*Escherichia*_phage_ArgO145	62,020	NC_049918	
V. cholerae B5-86	*Escherichia*_converting_phage_Stx2a_F451	64,900	NC_049924	[57]
V. cholerae Vc1	*Vibrio*_phage_Φ919TP	33,133	KU504502	[83]
V. gazogenes PB1	*Vibrio*_phage_VP882	38,197	NC_009016	[89]
	*Vibrio*_phage_martha_12B12	33,277	NC_021070	
V. nigripulchritudo TUMSAT-V. nig1	*Enterobacteria*_phage_mEp235	37,595	NC_019708	[90]
	*Sulfitobacter*_phage_pCB2047-2	—	—	
	*Enterobacteria*_phage_HK446	39,026	NC_019714	
V. nigripulchritudo TUMSAT-V. nig2	*Vibrio*_phage_PV94	33,828	NC_027368	[90]
V. nigripulchritudo TUMSAT-V. nig3	*Vibrio*_phage_PV94	33,828	NC_027368	[90]
V. parahaemolyticus N8-42	*Vibrio*_phage_K139	33,106	NC_003313	[47]
	*Vibrio*_phage_fs2	8,651	NC_001956	
V. parahaemolyticus N4-46	*Pseudomonas*_phage_D3	56,426	NC_002484	[47]
V. parahaemolyticus Q8-15	*Pseudomonas*_phage_D3	56,426	NC_002484	[47]
V. parahaemolyticus N1-22	*Vibrio*_phage_K139	33,106	NC_003313	[47]
V. parahaemolyticus B2-28	*Enterobacteria*_phage_N15	46,375	NC_001901	[91]
V. parahaemolyticus N2-5	*Enterobacteria*_phage_Mu	36,717	NC_000929	[91]
V. parahaemolyticus CHN25	*Vibrio*_phage_martha_12B12	33,277	NC_021070	[53]
	*Vibrio*_phage_VPUSM_8	34,145	NC_022747	
	*Vibrio*_phage_henriette_12B8	107,218	NC_021073	
	*Vibrio*_phage_N4	38,497	NC_013651	
V. parahaemolyticus S05	VPS05ph1	33,915	CP138328 (1034556-1068471)	[76]
	VPS05ph2	39,156	CP138329 (934020-973176)	
V. penaeicida IFO 15640T	*Vibrio*_phage_martha_12B12	33,277	NC_021070	[92]
	*Sulfitobacter*_phage_pCB2047_A	40,929	NC_020858	
	*Enterobacteria*_phage_mEp235	37,595	NC_019708	

	*Escherichia*_phage_ArgO145	62,020	NC_049918	
V. penaeicida IFO 15641	*Vibrio*_phage_martha_12B12	33,277	NC_021070	[92]
	*Enterobacteria*_phage_mEp235	37,595	NC_019708	
	*Escherichia*_phage_ArgO145	62,020	NC_049918	
	*Sulfitobacter*_phage_pCB2047_C	40,931	NC_020856	
	*Pseudomonas*_phage_phi2	41,871	NC_030931	
V. penaeicida IFO 15642	*Vibrio*_phage_martha_12B12	33,277	NC_021070	[92]
	*Enterobacteria*_phage_mEp235	37,595	NC_019708	
	*Escherichia*_phage_ArgO145	62,020	NC_049918	
	*Sulfitobacter*_phage_pCB2047_C	40,931	NC_020856	
	*Escherichia*_phage_TL-2011b	44,784	NC_019445	
V. penaeicida TUMSAT-OK1	*Vibrio*_phage_martha_12B12	33,277	NC_021070	[92]
	*Sulfitobacter*_phage_pCB2047_A	40,929	NC_020858	
	*Enterobacteria*_phage_mEp235	37,595	NC_019708	
	*Escherichia*_phage_ArgO145	62,020	NC_049918	
	*Pseudomonas*_phage_phi2	41,871	NC_030931	
	*Escherichia*_phage_TL-2011b	44,784	NC_019445	
	*Shigella*_phage_SfIV	39,758	NC_022749	
	*Shigella*_phage_POCJ13	62,699	NC_025434	
V. penaeicida TUMSAT-OK2	*Vibrio*_phage_martha_12B12	33,277	NC_021070	[92]
	*Enterobacteria*_phage_mEp235	37,595	NC_019708	
	*Escherichia*_phage_ArgO145	62,020	NC_049918	
	*Sulfitobacter*_phage_pCB2047_C	40,931	NC_020856	
	*Pseudomonas*_phage_phi2	41,871	NC_030931	
	*Shigella*_phage_SfIV	39,758	NC_022749	
	*Bacillus*_phage_vB_BtS_BMBtp14	50,740	NC_048640	
	*Salmonella*_phage_118970_sal3	77,375	NC_031940	
V. parahaemolyticus A1	*Vibrio*_phage_vB_ValM-yong1	33,851	NC_049477	[51]
V. parahaemolyticus A2	*Vibrio*_phage_VCY_phi	7,103	NC_016162	[51]
V. parahaemolyticus A5	*Vibrio*_phage_VCY_phi	7,103	NC_016162	[51]

V. parahaemolyticus A6	*Vibrio*_phage_Vf12	7,965	NC_005949	[51]
V. parahaemolyticus A7	*Vibrio*_phage_VfO3K6	8,784	NC_002362	[51]
V. parahaemolyticus A7	*Vibrio*_phage_VCY_phi	7,103	NC_016162	[51]
V. parahaemolyticus A7	*Enterobacteria*_phage_mEp043_c-1	42,780	NC_019706	[51]
V. parahaemolyticus A8	*Vibrio*_phage_VfO3K6	8,784	NC_002362	[51]
V. parahaemolyticus A8	*Vibrio*_phage_VCY_phi	7,103	NC_016162	[51]
V. parahaemolyticus A8	*Enterobacterial*_phage_mEp213	44,120	NC_019720	[51]
V. parahaemolyticus A9	*Vibrio*_phage_VfO3K6	8,784	NC_002362	[51]
V. parahaemolyticus A9	*Enterobacteria*_phage_Mu	36,717	NC_000929	[51]
V. parahaemolyticus A10	*Vibrio*_phage_VCY_phi	7,103	NC_016162	[51]
V. parahaemolyticus A10	*Vibrio*_phage_VfO3K6	8,784	NC_002362	[51]
V. parahaemolyticus A11	*Vibrio*_phage_VCY_phi	7,103	NC_016162	[51]
V. parahaemolyticus A11	*Vibrio*_phage_VfO3K6	8,784	NC_002362	[51]
V. parahaemolyticus A11	*Enterobacteria*_phage_mEp043_c-1	42,780	NC_019706	[51]
V. parahaemolyticus A12	*Vibrio*_phage_Vf12	7,965	NC_005949	[51]
V. parahaemolyticus A12	*Vibrio*_phage_VEJphi	6,842	NC_012757	[51]
V. parahaemolyticus A13	*Vibrio*_phage_VCY_phi	7,103	NC_016162	[51]
V. parahaemolyticus A13	*Vibrio*_phage_martha_12B12	33,277	NC_021070	[51]
V. parahaemolyticus A14	*Vibrio*_phage_martha_12B12	33,277	NC_021070	[51]
V. parahaemolyticus A14	*Vibrio*_phage_VfO3K6	8,784	NC_002362	[51]
V. parahaemolyticus A14	*Vibrio*_phage_VFJ	8,555	NC_021562	[51]
V. parahaemolyticus A15	*Vibrio*_phage_VfO3K6	8,784	NC_002362	[51]
V. parahaemolyticus A16	*Vibrio*_phage_VfO3K6	8,784	NC_002362	[51]
V. parahaemolyticus A17	*Vibrio*_phage_VFJ	8,555	NC_021562	[51]
V. parahaemolyticus A18	*Vibrio*_phage_VFJ	8,555	NC_021562	[51]
V. parahaemolyticus A19	*Vibrio*_phage_VFJ	8,555	NC_021562	[51]

V. parahaemolyticus A20	*Vibrio*_phage_VfO3K6	8,784	NC_002362	[51]
V. parahaemolyticus B1	*Vibrio*_phage_VfO3K6	8,784	NC_002362	[51]
V. parahaemolyticus B2	*Vibrio*_phage_VfO3K6	8,784	NC_002362	[51]
V. parahaemolyticus B3	*Vibrio*_phage_VFJ	8,555	NC_021562	[51]
V. parahaemolyticus B3	*Vibrio*_phage_VfO4K68	6,891	NC_002363	[51]
V. parahaemolyticus B4	*Vibrio*_phage_VCY_phi	7,103	NC_016162	[51]
V. parahaemolyticus B5	*Vibrio*_phage_VFJ	8,555	NC_021562	[51]
V. parahaemolyticus B7	*Vibrio*_phage_VFJ	8,555	NC_021562	[51]
V. parahaemolyticus B10	*Vibrio*_phage_VfO3K6	8,784	NC_002362	[51]
V. parahaemolyticus B11	*Vibrio*_phage_VfO3K6	8,784	NC_002362	[51]
V. parahaemolyticus VPE116	*Enterobacterial*_phage_mEp213<br>*Vibrio*_phage_VP882	44,120<br>38,197	NC_019720<br>NC_009016	[93]
V. parahaemolyticus VN-3218	vB_VpaI_VP-3218	11,082	LR595856	[80]
V. parahaemolyticus 10290	vB_Vipa10290	9,414	AVOH01000008 (1370845-1378511)	[41]
V. parahaemolyticus MAVP-26	*Vibrio*_phage_vB_Vipa26	10,893	MT188662	[41]
V. parahaemolyticus MAVP-36	*Vibrio*_phage_vB_Vipa36	9,721	MT188663	[41]
V. parahaemolyticus CDC_K5323G	vB_Vipa5323	—	MIUF01000019 (188973-190181)<br>MIUF01000072<br>MIUF01000065<br>MIUF01000002 (4141-6171)	[41]
V. parahaemolyticus CDC_K5308	vB_Vipa5308	—	MIUE01000114 (7376-8729)<br>MIUE01000031 (5201-8319)	[41]
V. parahaemolyticus 10–4255	*Vibrio*_phage_vB_Vipa71	10,295	MT193890	[41]
V. parahaemolyticus CDC_A8962	vB_Vipa8962	—	LHRO01000017<br>(70737-72090)	[41]
V. parahaemolyticus CDC_K5512	vB_Vipa5512	—	MIUZ01000070 (628-7658)	[41]
V. parahaemolyticus MEVP-10	*Vibrio*_phage_vB_Vipa10	9,318	MT188666	[41]
V. parahaemolyticus 1.146–15	phage_f237	8,784	AP000581	[41]

V. parahaemolyticus 1.220–16	ther	—	AAXNNA010000036	[41]
V. parahaemolyticus CTVP4291	*Vibrio*_phage_vB_Vipa4291	9,663	MT188665	[41]
V. parahaemolyticus MAVP-3	vB_Vipa3	—	NIXT01000011	[41]
V. parahaemolyticus MAVP-K	*Vibrio*_phage_vB_VipaK	10,809	MT188664	[41]
V. parahaemolyticus F30368	vB_Vipa30368	—	LRFV01000012 (755277-763577)	[41]

—: unavailable.

Table S2. The information of the selected 42 articles in this review.

Authors	Year of Publication	Sample Source	*Vibrio* Species	Reference
Chaguza, C.; Chibwe, I.; Chaima, D.; Musicha, P.; Ndeketa, L.; Kasambara, W.; Mhango, C.; et al.	2024	Clinical isolates	*V. cholerae*	[9]
Garin-Fernandez, A.; Wichels, A.	2020	Surface seawater collected from the North Sea from July 31st to August 5th, 2014	*V. parahaemolyticus* and *V. cholerae*	[26]
Steensen, K.; Séneca, J.; Bartlau, N.; Yu, X.A.; Hussain, F.A.; Polz, M.F.	2024	Coastal seawater collected in two close-by sampling locations namely Plum Island Sound Estuary, Ipswich, MA in 2007 and Canoe Grove, Nahant, MA in 2010	*V. cyclitrophicus*	[27]
Kobakhidze, S.; Koulouris, S.; Kakabadze, N.; Kotetishvili, M.	2024	NCBI nucleotide database	*Vibrio* spp.	[29]
Molina-Quiroz, R.C.; Dalia, T.N.; Camilli, A.; Dalia, A.B.; Silva-Valenzuela, C.A.	2020	—	*V. cholerae*	[30]
Chibani, C.M.; Hertel, R.; Hoppert, M.; Liesegang, H.; Wendling, C.C.	2020	Gut or gills of six different pipefish (*Syngnathus typhle*) caught in the same sea-grass meadow in Kiel Fjord in 2012	*V. alginolyticus*	[39]
Nawel, Z.; Rima, O.; Amira, B.	2022	—	*Vibrio* spp.	[40]
Foxall, R.L.; Means, J.; Marcinkiewicz, A.L.; Schillaci, C.; DeRosia-Banick, K.; Xu, F.; Hall, J.A.; Jones, S.H.; Cooper, V.S.; Whistler, C.A.	2024	Cinical and environmental isolates	*V. parahaemolyticus*	[41]
Santoriello, F.J.; Michel, L.; Unterweger, D.; Pukatzki, S.	2020	Clinical and environmental isolates	*V. cholerae*	[45]
Santoriello, F.J.; Pukatzki, S.	2021	—	*V. cholerae*	[46]

Xu, D.; Peng, X.; Xie, L.; Chen, L.	2022	*Paphia undulate*, *Perna viridis*, *Mactra veneriformis*, *Aristichthys nobilis*, *Carassius auratu*, and *Litopenaeus vannamei*	*V. parahaemolyticus*	[47]
Yu, L.H.; Teh, C.S.J.; Yap, K.P.; Ung, E.H.; Thong, K.L.	2020	Shrimp farms in Malaysia	*V. parahaemolyticus*	[49]
Wang, Z.; Wang, H.; Chen, D.; Li, Y.	2024	Seven shrimp aquaculture farms in Guangdong and Jiangsu provinces, China	*V. parahaemolyticus*	[51]
Mesa, C.A.D.; Mendoza, R.M.; Penir, S.M.U.; Peña, L.D. de la; Amar, E.C.; Saloma, C.P.	2023	*Penaeus monodon* from Masbate Island, Philippines	*V. harveyi*	[52]
Yang, L.; Wang, Y.; Yu, P.; Ren, S.; Zhu, Z.; Jin, Y.; Yan, J.; Peng, X.; Chen, L.	2020	Shrimps from fish markets in Shanghai, China	*V. parahaemolyticus*	[53]
Xu, Y.; Yang, L.; Wang, Y.; Zhu, Z.; Yan, J.; Qin, S.; Chen, L.	2022	Shrimps from fish markets in Shanghai, China	*V. parahaemolyticus*	[54]
Zhao, H.; Xu, Y.; Yang, L.; Wang, Y.; Li, M.; Chen, L.	2024	Shrimps from fish markets in Shanghai, China	*V. parahaemolyticus*	[55]
Li, X.; Wang, X.; Li, R.; Zhang, W.; Wang, L.; Yan, B.; Zhu, T.; Xu, Y.; Tan, D.	2023	Contamined sea cucumbers obtained from a hatchery in Liaoning Province, China	*V. alginolyticus*	[56]
Qin, X.; Yang, L.; Xu, Y.; Xie, L.; Wang, Y.; Chen, L.	2024	*A. nobilis*, *C. idellus*, and *P. pekinensis* from fish markets in Shanghai, China	*V. cholerae*	[57]
Wang, W.; Tang, K.; Wang, P.; Zeng, Z.; Xu, T.; Zhan, W.; Liu, T.; Wang, Y.; Wang, X.	2022	Gastric cavity of healthy *G. fascicularis* from Hainan Province, China	*V. coralliilyticus*	[58]
Rubio-Portillo, E.; Robertson, S.; Antón, J.	2024	Coral samples collected in June 2019 from the marine protected area of Tabarca in Spain	*V. mediterranei*	[59]
Li, X.; Zhao, L.; Gao, H.; Chen, L.; Fan, F.; Li, Z.; Fan, Y.; Li, J.; Liang, W.; Pang, B.; et al.	2021	Clinical isolates	*V. cholerae*	[65]
Pant, A.; Bag, S.; Saha, B.; Verma, J.; Kumar, P.; Banerjee, S.; Kumar, B.; Kumar, Y.; Desigamani, A.; Maiti, S.; et al.	2020	Clinical isolates	*V. cholerae*	[66]
Ochi, K.; Mizuno, T.; Samanta, P.; Mukhopadhyay, A.K.; Miyoshi, S.; Imamura, D.	2021	Clinical isolates	*V. cholerae*	[68]
Wang, H.; Yang, C.; Sun, Z.; Zheng, W.; Zhang, W.; Yu, H.; Wu, Y.; Didelot, X.; Yang, R.; Pan, J.; et al.	2020	Clinical isolates	*V. cholerae*	[70]
Hao, T.; Zheng, W.; Wu, Y.; Yu, H.; Qian, X.; Yang, C.; Zheng, Z.; Zhang, X.; Guo, Y.; Cui, M.; et al.	2023	Clinical and environmental isolates	*V. cholerae*	[71]
Behera, D.R.; Nayak, A.K.; Nayak, S.R.; Nayak, D.; Swain, S.; Maharana, P.K.; Biswal, B.; Pany, S.; Pati, S.; Pal, B.B.	2022	Clinical and environmental isolates	*V. cholerae*	[72]
Thong, K.L.; Tham, K.B.L.; Ngoi, S.T.; Tan, S.C.; Wan Yussof, W.N.; Ahmad Hanapi, R.; Mohamad, N.; Teh,	2022	Clinical isolates	*V. cholerae*	[73]

C.S.J.				
Wang, H.; Xie, G.; Huang, J.	2024	Shrimps from a farm in Hebei Province, China	*V. parahaemolyticus*	[76]
Soto, E.; Alegría, M.; Sepúlveda, F.; García, K.; Higuera, G.; Castillo, D.; Fontúrbel, F.; Bastías, R.	2024	NCBI nucleotide database	127 species	[77]
Nuidate, T.; Kuaphiriyakul, A.; Surachat, K.; Mittraparp-Arthorn, P.	2021	Dead shrimp in southern Thailand	*V. campbellii*	[78]
Garin-Fernandez, A.; Glöckner, F.O.; Wichels, A.	2020	Surface seawater collected from the North Sea	*V. parahaemolyticus*	[80]
Li, N.; Zeng, Y.; Hu, B.; Zhu, T.; Svenningsen, S.L.; Middelboe, M.; Tan, D.	2021	Chinese Center for Disease Control and Prevention	*V. cholerae*	[83]
Tan, D.; Hansen, M.F.; de Carvalho, L.N.; Røder, H.L.; Burmølle, M.; Middelboe, M.; Lo Svenningsen, S.	2020	Rainbow trout from Denmark	*V. anguillarum*	[84]
Xu, M.; Xu, M.; Tu, Q.	2021	NCBI nucleotide database	70 species and an unknown group	[87]
Qin, W.; Li, D.; Xu, L.; Lin, W.; Tong, Y.	2021	Crab farm in Zhejiang province, China	*V. alginolyticus*	[88]
Baby, B.; Vijay, D.; Philip, P.S.; Alnuaimi, A.A.; Almansoori, H.M.; Areidat, S.O.; Khan, G.; Vijayan, R.; Akhtar, M.K.	2023	Korean Collection for Type Cultures	*V. gazogenes*	[89]
Rathnapala, J.M.S.N.; Ragab, W.; Kawato, S.; Furukawa, M.; Nozaki, R.; Kondo, H.; Hirono, I.	2023	*P. vannamei* in a marine aquarium in Tokyo, Japan	*V. nigripulchritudo*	[90]
Yang, L.; Yu, P.; Wang, J.; Zhao, T.; Zhao, Y.; Pan, Y.; Chen, L.	2024	*Ruditapes philippinarum*, *Keenocardium californiense*, and *Oratosquilla oratori*	*V. parahaemolyticus*	[91]
Ragab, W.; Kawato, S.; Nozaki, R.; Kondo, H.; Hirono, I..	2022	*M. japonicus* in a shrimp farm in Okinawa, Japan	*V. penaeicida*	[92]
Zago, V.; Veschetti, L.; Patuzzo, C.; Malerba, G.; Lleo, M.M.	2020	Water in Caleri lagoon	*V. parahaemolyticus*	[93]
Garin-Fernandez, A.; Glöckner, F.O.; Wichels, A.	2020	The North Sea (station 1) on July 31st, 2014	*V. parahaemolyticus*	[80]

—: unavailable.
